# Combined Use of EMG and EEG Techniques for Neuromotor Assessment in Rehabilitative Applications: A Systematic Review

**DOI:** 10.3390/s21217014

**Published:** 2021-10-22

**Authors:** Cristina Brambilla, Ileana Pirovano, Robert Mihai Mira, Giovanna Rizzo, Alessandro Scano, Alfonso Mastropietro

**Affiliations:** 1 Istituto di Sistemi e Tecnologie Industriali Intelligenti per il Manifatturiero Avanzato (STIIMA), Consiglio Nazionale delle Ricerche (CNR), Via Previati 1/E, 23900 Lecco, Italy; cristina.brambilla@stiima.cnr.it (C.B.); robertmihai.mira@stiima.cnr.it (R.M.M.); alessandro.scano@stiima.cnr.it (A.S.); 2 Istituto di Tecnologie Biomediche (ITB), Consiglio Nazionale delle Ricerche (CNR), via Fratelli Cervi 93, 20054 Segrate, Italy; ileana.pirovano@itb.cnr.it (I.P.); alfonso.mastropietro@itb.cnr.it (A.M.)

**Keywords:** EMG, EEG, rehabilitation, neuromotor, evaluation, assessment, review

## Abstract

Electroencephalography (EEG) and electromyography (EMG) are widespread and well-known quantitative techniques used for gathering biological signals at cortical and muscular levels, respectively. Indeed, they provide relevant insights for increasing knowledge in different domains, such as physical and cognitive, and research fields, including neuromotor rehabilitation. So far, EEG and EMG techniques have been independently exploited to guide or assess the outcome of the rehabilitation, preferring one technique over the other according to the aim of the investigation. More recently, the combination of EEG and EMG started to be considered as a potential breakthrough approach to improve rehabilitation effectiveness. However, since it is a relatively recent research field, we observed that no comprehensive reviews available nor standard procedures and setups for simultaneous acquisitions and processing have been identified. Consequently, this paper presents a systematic review of EEG and EMG applications specifically aimed at evaluating and assessing neuromotor performance, focusing on cortico-muscular interactions in the rehabilitation field. A total of 213 articles were identified from scientific databases, and, following rigorous scrutiny, 55 were analyzed in detail in this review. Most of the applications are focused on the study of stroke patients, and the rehabilitation target is usually on the upper or lower limbs. Regarding the methodological approaches used to acquire and process data, our results show that a simultaneous EEG and EMG acquisition is quite common in the field, but it is mostly performed with EMG as a support technique for more specific EEG approaches. Non-specific processing methods such as EEG-EMG coherence are used to provide combined EEG/EMG signal analysis, but rarely both signals are analyzed using state-of-the-art techniques that are gold-standard in each of the two domains. Future directions may be oriented toward multi-domain approaches able to exploit the full potential of combined EEG and EMG, for example targeting a wider range of pathologies and implementing more structured clinical trials to confirm the results of the current pilot studies.

## 1. Introduction

Neuromotor disorders are developmental or acquired conditions usually caused by neurological diseases affecting the central nervous system that typically impair movement, gross and fine motor ability, and posture. It was recently reported that neurological disorders are the third most common cause of disability and premature death in the European Union [1], and their prevalence will increase with the progressive aging of the population. Therefore, neuromotor disorders associated with neurological diseases currently represent a burden for patients in all age ranges, health systems, and caregivers (formal and informal). A detailed comprehension of the processes underlying motor impairment, with direct involvement of the central nervous/peripheral nervous system, is at the basis of motor recovery understanding [2]. Moreover, the use of quantitative and instrumental neuromotor assessments can foster the design of effective therapeutic interventions and promote the development of personalized therapies to maximize motor recovery [3].

Different instruments and techniques have been developed to offer clinically reliable assessments of the neuromotor performances of patients. Two of the most valuable techniques used for analyzing, evaluating, and assessing motor performance employed in the rehabilitation field are EEG and surface EMG (sEMG, hereby only EMG). They record the electrical potentials that originated at cortical and muscular levels, respectively. EEG is a noninvasive and versatile technique that measures electrical activity related to neuron pools at the cortical level and is suitable for clinical, experimental, and real-life scenarios [4], whereas EMG measures the train of motor unit action potentials, generated by muscular contraction, through surface electrodes placed on the skin overlying muscle fibers [5]. They thus provide insights into neuromotor integrity/impairment by monitoring cortical activation and its motor correlates. Both techniques have been employed for neuromotor assessments, especially in research studies on rehabilitative applications.

In particular, EEG can explore the brain activity at the cortical and subcortical level and allows neuronal brain dynamics to be monitored with a high temporal resolution to explore whole-brain neuronal networks organization [6]. In the reference literature, EEG signal analysis was historically used in different applications to assess the activation pathways and to understand basic mechanisms underlying motor functions [7]. This led to more recent specific studies focused on neuromotor rehabilitation for investigating how activity patterns change depending on the location of the cortical lesions and on different rehabilitation treatments in different diseases such as stroke [8], Parkinson’s disease [9], and others. The evolution of the EEG signal analysis during the last decades shifted the focus from the time domain to the frequency domain analysis [10] with the more recent use of functional and effective connectivity approaches [11] to better understand neural network changes occurring in physiological and pathological conditions. Another relevant application is related to the use of EEG signal for interactively guiding the rehabilitation session using brain-computer interfaces (BCI) or biofeedback methods to control a rehabilitation robot [12] or a lower limb exoskeleton [13], to monitor the status of recovery [14] and to evaluate the patient’s engagement in traditional motor rehabilitation [15] and in virtual reality environments [16].

Analogously, in the rehabilitation field, EMG analysis has been used for a variety of assessments. Current applications of EMG are mainly related to the physiological investigation, monitoring of neurological disorders, and planning of treatments [17]. The study of muscle activity and coordination patterns is a useful tool for the identification of motor disorders and the evaluation of motor recovery after rehabilitation. Muscle activation patterns were identified in both upper [18] and lower limbs [19]; furthermore, EMG was employed for studying abnormal muscular activity, such as spasticity [20], and effect such as muscle fatigue [21]. Moreover, the factorization of the EMG signal is also at the basis of motor control theories, such as muscle synergies, which provide insight into the control mechanisms for motor planning [22,23]. Applications of this theory have been oriented toward quantifying motor control abnormalities [24] and changes in the muscular activation patterns [25]. EMG signals are also used to control exoskeletons for improving motor rehabilitation and to support daily life activities [26]. Interesting applications were also found in prosthetic control for amputees using residual EMG near the amputated region [27].

However, the employment of EEG and EMG signals has been only partially explored so far, especially in combined applications, although their coupling seems natural and effective [28]. It is indeed clear from existing literature that these techniques carry critical and complementary information regarding several aspects related to neuromotor assessment. In fact, it has been shown that these techniques allow a better understanding of pathologies involving the central nervous systems causing motor deficits, especially from the neuromotor point of view. EEG and EMG combined usage also contributes with detailed insights to the customization and tailoring of therapies by supporting the clinicians with relevant data on motor organization. Another potential impact provided with EEG and EMG is the outcome prediction. This issue has been explored with EEG [29] and EMG [30] in separate studies and acquires variability in a scenario that is evolving toward rationalization of the resources, containment of the costs, and rehabilitation efficiency [31].

Interestingly, we noticed that, despite their potential, EEG and EMG have been considered simultaneously in applications with assessment aims only occasionally. They might help in profiling the level of disability with multi-parameters approaches [32] and can constitute solid bases for novel approaches based on detailed multimodal assessments [33,34]. We also noticed a lack of comprehensive reviews describing which scenarios have been explored, what applications, setups, methods of analysis, and potential developments can be foreseen for such techniques, whereas most of the works where EEG and EMG are coupled focuses on brain-computer and multimodal interfaces for feedback and control [35]. Indeed, BCI and biofeedback are the first research fields for which the combination of the two signals has been successfully employed, and the literature of the past few years focused on these applications, providing an overview of the possibilities offered by the techniques until now.

Following the previous considerations, this systematic literature analysis aims to cover a field that has been less exhaustively described, reviewing all available studies in which EMG and EEG were combined for clinical practice, targeting applications of the two combined techniques not only for guiding rehabilitation but mostly for the evaluation and the assessment of physio-pathological motor function in both healthy subjects and patients. This review also provides critical comments on the current state-of-the-art approaches and future trends and directions.

## 2. Materials and Methods

This review attempts to answer the main research question (RQ 0): “How have EMG and EEG been combined in clinical practice for assessment of people in rehabilitation?”. RQ 0 is furtherly split into the following research questions:

RQ (1) Which type of experimental study design was employed?

RQ (2) Which groups of subjects, pathologies, and anatomical segments were targeted with the combined EEG-EMG approach in rehabilitation?

RQ (3) What setups were used for rehabilitation and signal acquisition?

RQ (4) What analysis techniques have been employed and what results were achieved?

We thus considered papers that applied EMG and EEG simultaneously provided an overview of which scenarios were considered for applications and which setups were used for rehabilitation and acquisitions, explored the data analysis techniques and the achieved results. The international guidelines established by PRISMA (Preferred Reporting Items for Systematic Reviews and Meta-Analyses) [36] were used.

### 2.1. Criteria for Papers Classification

Our review of the previous literature was organized to summarize the state-of-the-art of the field by detailing the following categories:

- TYPE OF STUDY: lists the papers based on the study type described in the text (e.g., Observational, Pilot, Randomized Controlled Trial, and Methodological).

This section answers the question RQ 1.

- SUBJECTS AND ANATOMICAL TARGETS: describes to which clinical scenarios EEG and EMG were applied together. This section answers the question RQ 2.

This section was further specialized into:

*Cohort of Subjects:* aiming to summarize what kind of subjects were enrolled for combined EEG-EMG studies (e.g., post-stroke subjects and healthy controls) and what sample size of patients/subjects was enrolled in the experimental studies.

*Anatomical Targets:* aiming to review which anatomical segments were assessed and/or rehabilitated in concurrent EEG-EMG studies (e.g., upper limb).

- EXPERIMENTAL SETUP and PROTOCOLS: describes which experimental setups were employed for rehabilitation and data acquisitions. This section answers the question RQ 3.

Experimental setup and protocols were further divided into:

*Setup for tests/rehabilitation:* aiming to describe which setup was used for rehabilitation (e.g., robotic assistive device)

*Setup for signal acquisition:* aiming to review what setups were used for data collection (e.g., 16 channel s-EMG).

- DATA ANALYSIS: describes which techniques and findings were used for data analysis. This section answers the question RQ 4.

Data analysis was further divided into:

*Analysis Techniques:* aiming to describe which techniques have been employed and which domains and features were considered in the analysis (e.g., time/frequency).

*Benefits of combined EEG-EMG applications:* aiming to describe which were the main findings that were achieved using the combined EEG-EMG acquisition and analysis.

### 2.2. Bibliographic Research Criteria

With the above-mentioned aims, the following procedure was employed for the literature screening. A collection of articles was obtained by screening PubMed, Scopus, and Web of Science (WOS), using a query based on the keywords: “EEG”, “EMG”, “MUSCL*”, “MOTOR*”, “MOVEMENT”, “MOTION”, “REHABILITATION” and excluding the keyword “BCI”. Articles strictly concerning BCI and biofeedback implementation with electrical biological signals were excluded since their main aim is commonly not focused on combined EEG/EMG functional assessment.

The formal logical query was (EEG) AND (EMG OR MUSCL*) AND (MOTOR* OR MOVEMENT OR MOTION) AND (REHABILITATION) AND NOT (BCI).

### 2.3. Eligibility Criteria

In the eligibility phase, we distinguished the papers relevant to the aim of this review. For being eligible, screened papers had to satisfy all the following criteria:

(A) To include the specified query in the abstract and/or title and/or in the keywords
(B) To involve the simultaneous use of EMG and EEG
(C) EMG and EEG had to be used for neuromotor assessment
(D) To target rehabilitation scenarios
(E) To be indexed in at least one of the screened databases
(F) To be a full article (at least 4 pages)
(G) To be available in English

The papers were screened, one by one, for inclusion by two different groups (composed of subgroups of the authors of the paper) independently. The main inclusion criteria had to include: “criteria A AND B AND C AND D AND E AND F AND G”. Each paper was screened by two different reviewers who blindly classified it as eligible or non-eligible. Any disagreement in the classification was settled by discussion between the two groups, and a consensus was reached in all cases.

## 3. Results

### 3.1. Selected Papers

**Figure 1:**
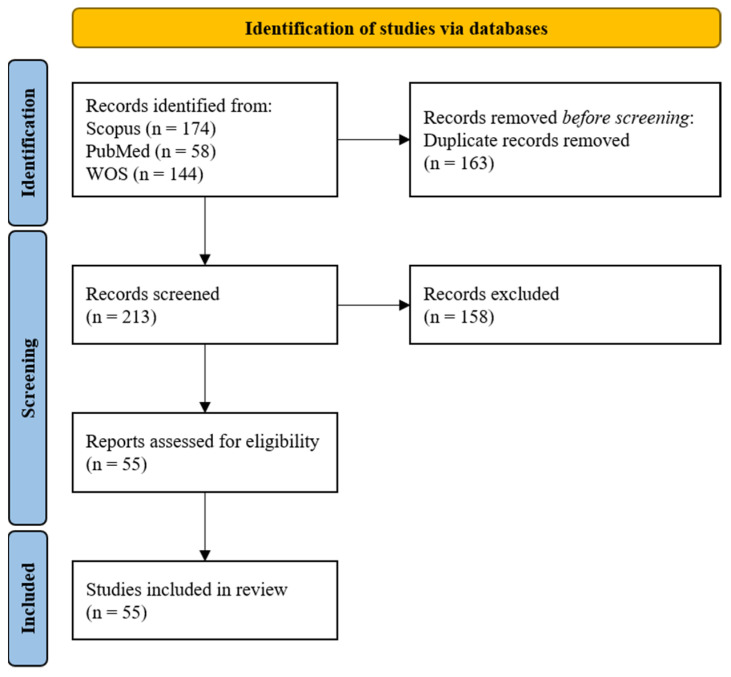
The PRISMA flow chart for the proposed literature review [36].

As a result of the screening, 174 papers were found on Scopus, 58 on PubMed, and 144 on WOS. The total number of articles was 385. Out of all these articles, 163 were duplicates across the 3 databases. The number of studies eligible for the detailed screening was 213. After the screening phase, the number of papers identified as eligible, meeting all the selection criteria, and included in the review was 55. In the next sections, the results of our research are presented. The PRISMA flow chart summarizing all the steps for screening and inclusion is presented in Figure 1.

**Figure 2:**
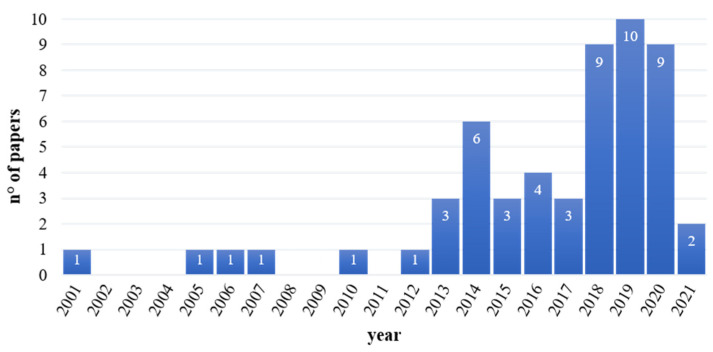
Temporal distribution (number of papers published per year) of selected papers.

As shown in Figure 2, most of the papers describing concomitant applications of EEG and EMG in the assessment of neuromotor skills in rehabilitation were recently published. Indeed, more than 50% of the papers included in this review were produced in the last 5 years.

**Figure 3:**
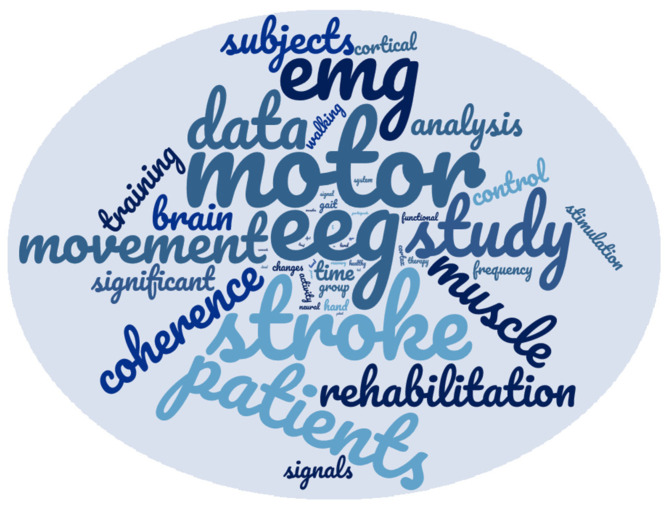
Word-cloud representing the 50 most cited words (excluding all the words not representing nouns and not relevant acronyms) included in the text of the papers selected in this review. The higher the size, the higher the number of citations inside the papers.

An exploratory analysis of the 50 most cited words within the selected papers has highlighted that they appear overall 34,540 times in the text, and they represent more than 10% of all the words composing the whole documents. Among them, the most cited word is motor (1900 citations), followed by EEG (1845) and stroke (1805). Among the first 10 most cited words, we also found: EMG, patients, study, data, movement, muscle, and coherence. A pictorial representation is shown in Figure 3.

### 3.2. Type of Study

**Figure 4:**
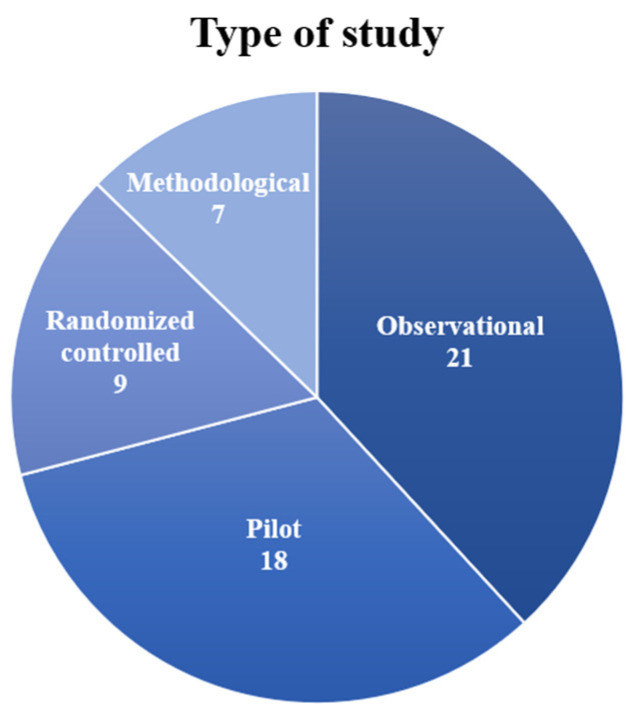
Pie chart portraying the number of selected studies for each study design (Observational, Pilot, Randomized Controlled, Methodological).

In this section, the papers were subdivided according to the study design proposed by the experimenters. In Table 1, we grouped the works into four categories: observational study, pilot study, randomized controlled trial, and methodological study. For each paper, we also detailed the aim of the study. The distribution of papers in the categories is shown in a pie chart in Figure 4.

A total of 21 papers out of 55 (37%) presented observational studies in which functional parameters or effects of treatments were investigated on healthy subjects and patients. An aim commonly found in these works was the assessment of the cortico-muscular coupling during movements [37,38,39,40,41] as a method to better understand motor control mechanisms for improving the rehabilitation design. Cortico-muscular coherence was also tested as a tool for investigating the effects of functional electrical stimulation [42,43,44,45,46]. Some studies analyzed the effects of treatments based on exoskeletons on neuromotor outcomes [47,48,49]. The efficacy of visual feedback was assessed to explore novel rehabilitation paradigms [50,51]. Two studies only [52,53] were interested in detecting movement intention coupled with EEG and EMG recordings. Other works investigated specific parameters typical of each study: Palmer et al. [54] studied the interhemispheric interaction using transcranial stimulation, Vladimirov et al. [55] searched neurophysiological markers of stress, Yilmaz et al. [56] investigated slow cortical potentials in stroke patients, and Jacobs et al. [57] studied the correlation between low back pain and postural stabilization.

Papers that tested a novel experimental setup or concept design on a limited number of subjects were classified as pilot studies. A total of 18 out of 55 papers (33%) were classified as pilot studies. Many of these studies presented novel rehabilitation paradigms based on robotics [58,59,60] or exoskeletons [34,61,62]. Donati et al. [63] tested a multi-stage brain-machine interface (BMI), while Hashimoto et al. [64] used the EEG feedback for improving rehabilitation. Some studies presented preliminary results for methods of movement classification [65], detection of movement intention [66,67], and motor imagery [68]. Three studies investigated cortico-muscular coupling [69,70,71] as a novel method to evaluate the motor recovery of post-stroke patients. Neuroplastic changes induced by TMS were studied by Dutta et al. [72] to improve rehabilitation technologies. Moreover, pilot studies were conducted for the evaluation of the level of engagement during game rehabilitation [73] and of the effects of virtual reality on facial rehabilitation [74].

A total of 9 studies out of 55 (17%) presented randomized controlled trials that subdivided the enrolled cohorts into treatment groups compared to the control groups to test the validity of an experimental setup. Bao et al. [75] and Benninger et al. [76] studied the efficacy of employing transcranial stimulation on stroke and parkinsonian patients, respectively, while three studies applied peripheral electrical stimulation on stroke patients [77,78,79]. Rehabilitation for stroke patients was investigated by Calabrò et al. [80] using an exoskeleton and by Chen et al. [81] with a novel treadmill. Furthermore, the efficacy of sensorial feedback based on music [82] or EEG/EMG biofeedback [83] in stroke patients was assessed during rehabilitation to improve motor recovery.

Finally, seven papers (13%) were classified as methodological studies: they presented novel methods and algorithms for analyzing together EEG and EMG signals. Cisotto et al. [84] provided a method for compressing EEG and EMG signals. Other studies developed algorithms for detecting motion [85,86] and classifying it [87,88]. Belfatto et al. [32] and Pierella et al. [33], instead, showed a methodology for a multivariate motor assessment aiming at proposing a novel methodology for evaluating rehabilitation.

### 3.3. Subjects and Anatomical Targets

This paragraph describes the properties of the cohorts of subjects involved in the experimental sessions considering the clinical status of the subjects (healthy vs. pathologic), age range, and the sample size. Furthermore, the anatomical targets for the functional assessment and/or rehabilitation are described. A summary of the most relevant results described in this section is reported in Table 2.

#### 3.3.1. Cohorts of Subjects

In the papers analyzed in this review, the cohorts of subjects involved during the experimental sessions could be divided into two macro-categories: (i) healthy subjects and (ii) patients affected by different diseases and pathological conditions affecting the neuromotor system. In particular, 36 out of 55 (64%) studies have enrolled healthy volunteers as either target groups (19 out of 36–53%) or control groups (17 out of 36–47%). Conversely, 36 out of 55 (65%) papers have enrolled patients.

Most of the studies involving patients were focused on stroke (23 out of 36–64%) at different stages. The chronic stage was studied in 20 out of 23 papers (87%), the subacute phase was described in 1 paper (4%), whereas a longitudinal analysis (subacute and chronic stage) was performed in 2 out of 23 papers (9%).

**Figure 5:**
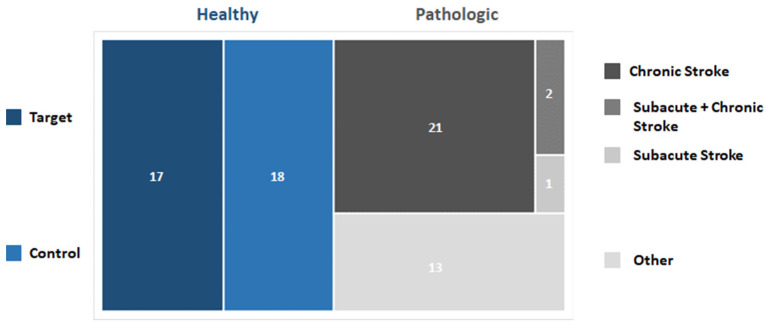
Graph representing the distribution of selected papers based on the cohort of subjects enrolled.

A total of 13 out of 36 papers considered other types of pathological conditions such as: spinal cord injury (4 out of 13–31%), mixed injuries and diseases (2 out of 13–15%), Parkinson’s disease, cerebral palsy, writer’s cramp, low back pain, facial palsy, mild cognitive impairment and cardiovascular diseases (1 document each). See Figure 5 for a schematic representation of the results.

The age of the subjects involved in the studies ranged from 5 to 92. Most of the healthy subjects’ cohorts were composed of young adults (up to 40) (21 out of 36 documents—58%), whereas the patients’ cohorts were mostly composed of adults (> 40) or older adults. (>65) (29 out of 36 papers—80%).

**Figure 6:**
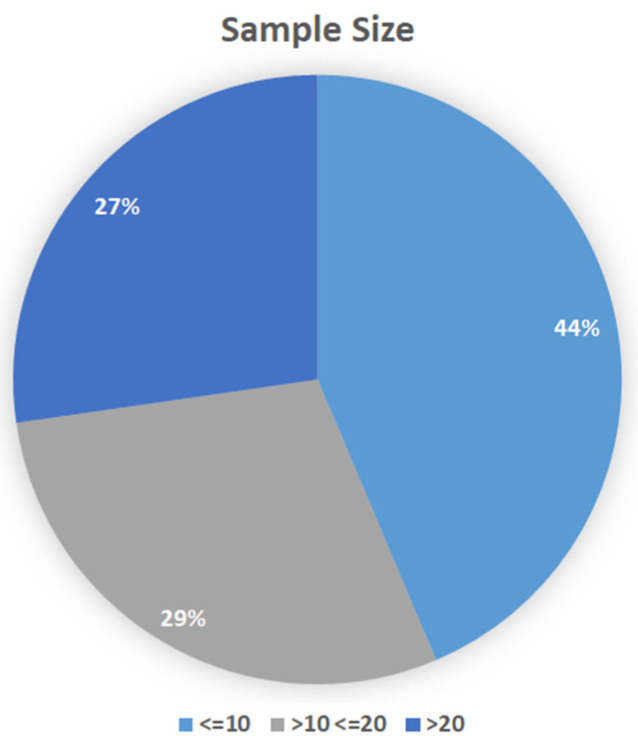
Pie chart portraying the percentage of selected studies for the sample size.

Considering the sample size, 24 out of 55 (44%) papers involved at most 10 subjects, 16 out of 55 papers (29%) enrolled up to 20 subjects, whereas 15 out of 55 papers (27%) enrolled more than 20 subjects. It is worth noticing that four papers describe results based on a single subject analysis, whereas the highest number of subjects involved was 42. See Figure 6 for a schematic representation of the results.

#### 3.3.2. Anatomical Targets

Regarding the anatomical regions that were objects of study, the distal upper limb was predominantly considered in 24 out of 55 papers (44%), the proximal upper limb was evaluated in 21 out of 55 documents (38%), the distal lower limb is included in 13 out of 55 papers (24%) whereas the proximal lower limb in 8 out of 55 papers (15%). It is worth noticing that in 3 out of 55 documents (5%), the focus was put on other regions (torso, face, neck).

Specifically, considering the distal upper limb, most of the applications were focused on the assessment/rehabilitation of hand movements (14 out of 24 papers—58%); 5 out of 24 studies (21%) considered the wrist, whereas 5 out of 24 (21%) documents described the concurrent analysis of wrist and hand. As to the proximal upper limb, 9 out of 21 (43%) papers were focused on the elbow, 3 out of 21 (14%) generically on the arm, 1 out of 21 (5%) on the shoulder, whereas 8 out of 21 (38%) studies were focused on the combined analysis of shoulder and elbow.

**Figure 7:**
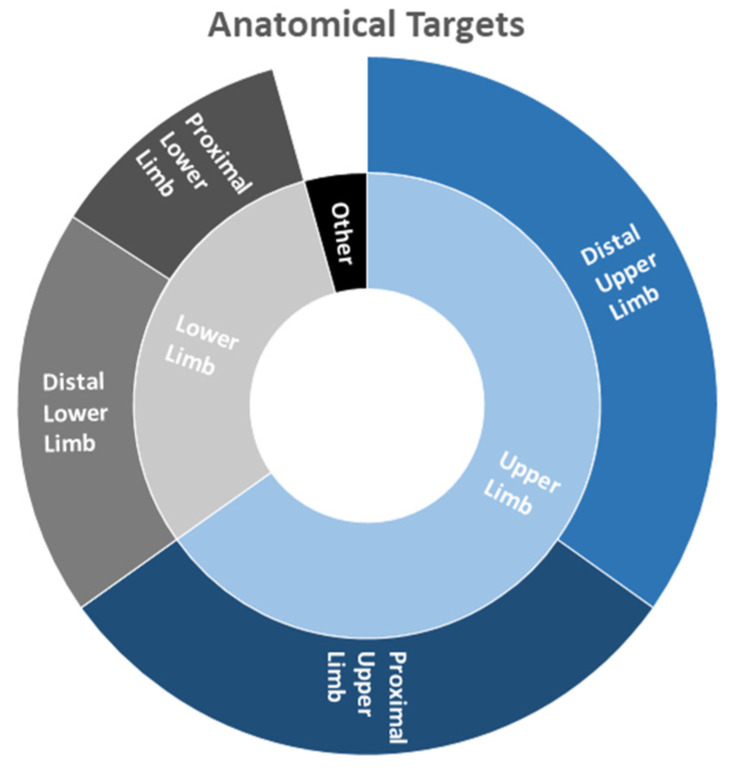
Hierarchical representation of anatomical targets considered in the analyzed documents.

As to the lower limb, in the distal part, 7 out of 13 (54%) papers focused on the ankle, 5 out of 13 (38%) documents on the leg, and 1 out of 13 (8%) studies on the foot movements. Finally, considering the proximal lower limb, 6 out of 8 (88%) papers focused on the knee whereas 1 out of 8 (12%) on the simultaneous analysis of hip and knee. A schematic representation of the results is shown in Figure 7.

### 3.4. Experimental Setups and Protocols

The screened papers were subdivided according to the setup employed for the rehabilitation and/or assessment. The main categories reported in Table 3 were identified as miscellaneous techniques for free movement and rehabilitation, robotic assistance, peripheral electrical stimulation, transcranial electrical stimulation, and assisted rehabilitation. Papers presenting techniques possibly ascribable to multiple categories were assigned to the best fitting one.

Among the selected studies, we grouped studies addressing miscellaneous techniques in the group “Miscellaneous techniques for free movement rehabilitation”. In this category, we selected papers that did not include aids, robots, and supports or that used devices that are very typical of a specific study or do not belong to a specific category. Some studies assessed the motor function only with simple movements performed by subjects, such as wrist [69,84], elbow [38], arm [37,57,70,71,87,88], hand [40,67,83,86] and leg tasks [53] and respiratory movements [55]. Additional sensorial feedbacks were employed for evaluating the effects of visual [65,73], auditory [82], and audiovisual feedback [56]. In one paper, the additional neurofeedback allowed the improvement of functional recovery of the hand in dystonic patients [64]. Moreover, in two studies [53,85], movements were compared to motor imagery. Bartur and colleagues [50] employed mirror visual feedback in the rehabilitation setup for hemiparetic stroke patients. The effectiveness of a novel balance handle was assessed with arm movements [52].

Robotic solutions and exoskeletons were employed in 16 papers for motor rehabilitation. Exoskeletons for lower limbs were present in six studies and were tested in walking tasks [47,48,80], on a treadmill [49], and in virtual reality environments [34,63]. One study only [41] presented a mobilizer specific for the ankle. In the selected papers, exoskeletons for upper limbs were designed for hand mobilization and finger movements [39,61,62,66]. Four papers [32,33,58,59] presented a robotic end effector for the upper limb, and Park et al. [60] developed a robotic mirror therapy for the arm.

For improving motor recovery, peripheral stimulation, in which the stimulus is applied to the nerve to induce the contraction of the muscles, was employed in different studies. Functional electrical stimulation (FES) is a popular technique, and it was used for stimulating hand muscles [43,45,46,78] and ankle muscles [79]. Other techniques for peripheral stimulation are endogenous paired associative stimulation (ePAS), used by Olsen et al. [77] for the ankle joint muscles, and neuromuscular electrical stimulation (NMES), employed by Xu et al. [44] for wrist muscles. In transcranial electrical stimulation, instead, the electrical stimulus is delivered at the cortical level through electrical current, as in transcranial direct current stimulation (tDCS), or magnetic field, as in transcranial magnetic stimulation (TMS). Bao et al. [75] presented a high-density tDCS associated with wrist contractions for stroke rehabilitation, while Dutta et al. [72] used an anodal tDCS for assessing neuroplastic changes. Palmer et al. [54] employed TMS for studying the cortico-muscular coherence in stroke patients, while Benninger et al. [76] assessed the safety of using repetitive TMS for treating parkinsonian symptoms.

In the assisted rehabilitation group, we collected experimental setups in which devices that helped the movements performed during rehabilitation were included. In Bao et al. [42], a pedaling system coupled with NMES was employed for motor rehabilitation in stroke patients. A novel turning-based treadmill was presented by Chen et al. [81], while Jensen et al. [51] added the visual feedback to rehabilitation on a motorized treadmill.

Very few papers included virtual reality (VR) environments in rehabilitation: VR environment alone was present in only one study [74], while Bulea et al. [34] and Donati et al. [63] used virtual reality in combination with exoskeletons.

### 3.5. Setups for Signal Acquisition

We analyzed the setup used for the acquisition of EEG and EMG signals according to the type of system, the number, and the positioning of the electrodes. The electrode positioning of EEG was related to the area of the brain from which the signal was recorded and was identified in the motor area only, sensorimotor area, and whole cortex; for EMG signal, we subdivided the positioning based on the number of joints and limbs involved: therefore, we sorted papers in single-joint, multi-joint and multi-limb. The details are reported in Table 4, divided into EEG and EMG acquisition setups. Unfortunately, not all the papers declared the systems used in detail; therefore, we reported only the studies in which the recording systems were clearly specified.

For the EEG signal, different types of systems were employed for the signal acquisition, and all the instruments were commercial systems, reported in Table 4. Two papers only used customized amplifiers [51,86]. We noticed that no system was preferred with respect to the others, but a variety of instruments were employed in the literature. In addition, the set sampling frequency and the impedance were different among the studies.

The number of EEG channels changed depending on the study design: a higher number of electrodes was used for a more comprehensive mapping of the whole cortical area, while a lower number gives details on the activity of specific areas. Five papers employed more than 100 electrodes for recording EEG signals: 163 electrodes were used for studying cortico-muscular coupling [70,71], 160 for mapping brain activity [58], and 128 for neurophysiological assessments [42,59].

Many papers employed the standard number of electrodes that are usually provided with EEG caps: nine studies used 64 electrodes [32,33,34,38,44,50,65,66,75], six used 32 electrodes [40,52,53,54,81,84], five used 16 electrodes [43,49,56,63,78] and one applied 8 electrodes [83]. No standard number of electrodes was found in the other papers. Li et al. in two studies [47,48] employed 62 electrodes, Mima et al. [37] used 56 electrodes, Olsen et. al. [77] and Yang et al. [68] applied 40 electrodes and Lou et al. [67] used 35 channels. Other studies recorded the EEG signal with a lower number of electrodes: 21 electrodes were used in two papers [39,80] and 20 in other two [69,82]; six studies employed between 15 and 10 electrodes [57,61,62,64,73,85].

Finally, some papers applied very few electrodes: Dutta et al. [72] used five channels, four studies used three electrodes [60,86,87,88], and Zhai et al. in two studies [45,46] employed only two electrodes. Four studies [41,51,55,79] applied only one channel for recording EEG signal: the electrode was positioned in Cz of the 10–20 electrode placement system in all these studies.

Papers that employed a high number of electrodes recorded the whole cortical activity with a high density of probes, but other papers acquired the activity of the whole cortex also with a lower number of probes [62,63]. Usually, few electrodes were employed for recording the activity of the sensorimotor area [39,43,60,64,78] or the motor area only [41,61,67].

As for the EEG acquisition setups, the types of systems employed for EMG signal were different among all the studies, and two papers only customized the setups [42,86], while all the other used commercial systems, reported in Table 4. Moreover, the sampling rate and the impedance used for the recording were different among the studies.

Many studies applied few electrodes for recording the activity of a single muscle or a pair of agonist and antagonist muscles: 4 electrodes were used in 12 studies, 3 in 5 studies, 2 electrodes were employed in 5 papers, and a single probe was used in 12 papers. Using a few electrodes, the activity was recorded from muscles controlling only one joint. The employment of more probes allows recording the activity of some muscles that underlie multi-joint coordination: three papers [57,63,84] used five electrodes, two papers [40,49] six electrodes and six studies applied eight electrodes [32,56,70,73,76,80]. Finally, the EMG activity was recorded from more than 10 electrodes in 6 papers [33,37,53,58,62,71].

The EMG electrode positioning was classified based on the number of joints that are controlled with the muscles recorded. The upper limbs were investigated more than the lower limbs in both single and multiple joint categories. The wrist joint was studied the most: 17 papers analyzed muscles of the forearm moving the hand. Seven studies, instead, recorded muscles that move the whole upper arm. Two papers [34,72] placed the EMG probes on knee muscles, while muscles moving the ankle joints were recorded in four papers [41,51,77,79]. One study [74] acquired the activity of facial muscles. Studies that employed a higher number of EMG electrodes recorded the activity of muscles that regarded more than one joint: 7 papers analyzed the muscular activity of the lower limb, 12 studied the upper limb, and Jacobs et al. [57] included the trunk analysis to support the upper limb one. Investigating more joints allows the analysis of muscle activation patterns and muscle synergies, giving an insight into motor control [23]. Only four studies [37,70,71,76] involved both upper limbs in the EMG acquisitions.

### 3.6. Data Analysis

#### 3.6.1. Analysis Techniques

In literature, various techniques are employed to analyze EEG and EMG signals accordingly to the different aims of the specific studies. In Table 5, we identified five different macro-categories of analysis most frequently used for EEG and EMG independently. Three further categories of combined EEG-EMG analysis were identified. For each macro-category, a further detailed sub-classification was made, based on specific approaches implemented. Frequently, more than one technique was employed in the same study. The list of papers reported in Table 5 was sorted accordingly as follows.

**Figure 8:**
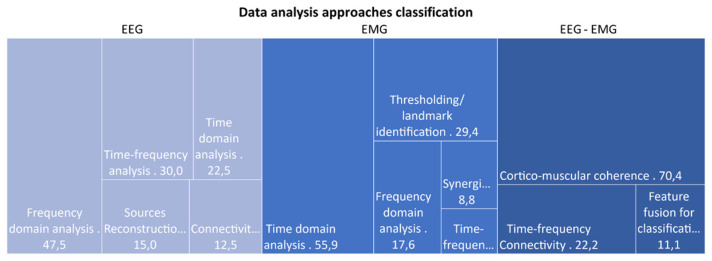
Graphical representation of data analysis approaches categories, divided according to the signal considered: only EEG, only EMG, or EEG and EMG combined. Percentage of papers using features belonging to each specific category are reported in the graph.

Among the 55 papers selected, we found that 72.7% of them analyzed the EEG signal individually, while 61.8% performed an analysis on the EMG signal alone. Only 49.1% of the papers extrapolated features combining EEG and EMG signals. In Figure 8, a representation of the different categories of found data analysis is provided, divided by the macro-categories identified in Table 5. For what concern the EEG analysis, we noticed that most of the papers (47.5%) exploited a frequency domain approach, while the time domain one is the most represented approach for the analysis of EMG signal (55.9% of EMG alone papers). Finally, the extraction of the cortico-muscular coherence (CMC) was the most widespread metric employed (70.4%) among papers that considered the EEG and EMG signals in combination.

Hereafter, we focus on the description of the identified macro-categories and on the features proposed in the literature.

In the first instance, the signals analysis can be classified based on the domain of feature extraction, i.e., in time, frequency, or time-frequency domain. In time domain approaches, EEG and EMG temporal series are directly analyzed after a pre-processing step to remove artifacts.

Only nine of the selected papers focused on the extraction of EEG features in the time domain: in five works, movement event-related potentials (ERP) were analyzed with respect to an external stimulus or a voluntary movement. In [53,74,87,88], specific features of the cortical electric potentials were considered, such as amplitude, slope, fractal dimension, and Hjorth parameters of the cortical response [89].

In contrast with EEG, time domain features are frequently extrapolated from the envelope of the EMG signal. A total of 19 papers implemented this type of approach (see Table 5). Specifically, 13 of them focused on the information extrapolated by the amplitude or the root mean square (RMS) of the envelope to quantify the muscles fibers’ activity, while in 6 works, additional time features were calculated. For example, Guo and colleagues [39], as well as Hashimoto and colleagues [64], considered the envelope integral. Tryon and colleagues [87,88] fitted the EMG experimental signal with an auto-regressive model, also calculating the mean absolute value, the mean absolute slope, the waveform length, and zero crossings. Moreover, Yao et al. [70] were able to define a muscle selection index from the temporal series of EMG electrodes.

Even though the time series can provide usual information on the biological processing underling the recorded signals, a complementary analysis can be performed in the frequency domain. The Fourier transforms of the temporal signals are calculated, and the spectral content at specific frequency bands is usually evaluated.

For what concerns the EEG analysis, 19 papers among the ones selected (second raw Table 5) employed this type of approach. Typically, in EEG, the power spectral density (PSD) averaged over epochs of the entire signal is calculated, and five spectral bands of interest are identified, i.e., delta (δ: 0.5–4 Hz), theta (θ: 4–8 Hz), alpha (α: 8–13 Hz), beta (β: 13–30 Hz) and gamma (γ: 30–150 Hz). The power amount in each of these bands and their ratio provides information on a particular mental state and cognitive involvement. Only in 4 of the 19 papers identified, specific quantitative bands power-based indexes were calculated, e.g., a relative amplitude value [60], an engagement index (β/(α + θ)) [62,73] and the θ/β ratio [55].

Only six papers exploited the frequency approach to investigate the EMG signal. As for EEG, in the work of [41,47,49], the EMG signal was segmented and the average PSD calculated, identifying responses at specific frequencies or spectral correlation between different muscles signals. In [44,60,79], a specific index, i.e., the median frequency, was calculated to investigate the occurrence of muscles fatigue during exercise.

Especially in the assessment of rehabilitation, it is important to evaluate the neuromotor response related to specific movement or intervention in time. The time-frequency domain analysis allows combining the spectral information retrieved from the EEG and EMG signals as they vary during time. This type of analysis has been proposed in EEG studies to evaluate the rise of cerebral waves at a different frequency. In particular, the event-related desynchronization/synchronization (ERD/ERS) is usually calculated as the percentage power decrease or increase at specific frequency bands following a movement onset. The ERD/ERS represents the synchronization or desynchronization of neuron populations in response to a voluntary muscle activation [7]. Among the selected, nine papers exploited this type of time-frequency analysis to explore the frequency-specific brain response over time. In three further studies [42,63,73], this approach has been extended in the evaluation of active/passive muscle stimulation during cycling or walking and brain-computer interface application, thus using the more general term event-related spectral perturbation (ERSP) to indicate the type of outcome obtained.

Also, for EMG, it is possible to exploit the conjunction between spectral and temporal information, even though this approach is not often used in the applications that we considered in this review work. In fact, only two papers exploited this analysis. Jensen in 2018 [51] performed a time-frequency-based analysis of the coherence between five EMG channels during a visually guided walking task. Li and colleagues [47] evaluated the correlation of EMG PSD of four channels in six frequency bands during the gait cycle.

As explained, an accurate time-frequency analysis of both EEG and EMG signals requires the identification and synchronization of the biological signals time series with the movement, or more in general to the experimental event of interest. Many studies reported in literature exploit the EMG signal for the exact timing of the onset of movement/experiment. In Table 5, we found 11 works in which EMG thresholding algorithms were employed, primarily to identify the onsets, thus leading the following analysis on both EEG and EMG signals.

The analysis approaches described until now usually considered the signal registered from each electrode (EEG or EMG) independently. However, a second-level analysis can be performed, taking into consideration not only the temporal or frequency information but also their spatial distribution and connection. For the cerebral signal, this approach includes connectivity analysis among brain areas, while for EMG, muscle synergies represent spatial patterns involving the recruitment of multiple muscles. Synergies are coordinated activations of groups of muscles as a consequence of a common control signal from the central nervous system [90]. We identified three papers [32,33,53] that implemented this type of investigation in rehabilitation assessment, exploiting the non-negative matrix factorization algorithm [91].

Brain connectivity analysis aims to identify those areas that are synchronously active both at rest and during a specific task. Two types of approaches can be distinguished: functional and effective analysis. In functional analysis, the functional network organization is investigated; in effective analysis, also the directionality and the causal influence between structures are evaluated [92]. We found two works exploring the EEG functional brain connectivity [81,85] and three papers quantifying the effective connectivity [48,54,80] to investigate the effect of rehabilitation or intervention on a patient’s cortex connections reorganization.

The EEG analyses can be performed either by directly analyzing data on the electric potential difference registered at each electrode or, as an alternative approach, an intermediate step of reconstruction of the cortical sources can be added to retrieve the temporal and spectral series of the generators of the brain electric field. In our review, we found that most studies were conducted exploiting the electrode signals directly, and only in six papers (see table) the reconstruction of sources was performed. Among these, two different main approaches were used: the first one based on independent component analysis (ICA) of the electrodes signal and fitting of the dipole model [93]; and the second one based on the low-resolution brain electromagnetic tomography (LORETA) [94].

Even though all the papers selected in this literature review combine the acquisition of EMG and EEG, authors often conclude with a separate analysis of the two signals and a combined observation of the results. Only in 28 over 55 works a quantitative combination metric of EEG and EMG was considered. Mostly, the cortico-muscular coherence (CMC), defined as the coherence function between the EEG and EMG signals, was quantified [95] in 16 works. In [42,46], the extended concept of partial directed coherence (PDC) [96] and generalized PDC (gPDC) [97] was applied to identify also causal information in CMC. Six studies also explored the application of time-frequency connectivity methods for the investigation of the relation between muscular and cerebral electrical signals. Cremoux et al. [38], as well as Jensen et al. [51], exploited a wavelet cross-spectrum-based approach, while Chen et al. [81] and Kim and colleagues [66] employed the cross-mutual information metric. In [47], Pearson’s correlation coefficients between EEG and EMG channels were calculated. In [40], an effective connectivity method was implemented based on Copula Granger’s causality.

Finally, the studies by Leerskov [65] and Tryon and colleagues [87,88] must be mentioned since they pointed out two different classification approaches for the classification of motion and control of robotic rehabilitation devices through the fusion of features derived from EEG and EMG.

#### 3.6.2. Benefits of Combined EEG-EMG Applications

In some papers included in this review, EMG has an ancillary role with respect to EEG since it was used to synchronize EEG signals with respect to relevant functional events composing the experimental/rehabilitation protocol (e.g., target movements, cognitive stimuli, electrical muscle stimulations, etc.). More interestingly, there are papers that combine the EEG/EMG signals to extract new relevant combined features. For example, the use of CMC can help to detect voluntary movements in spastic subjects or can be used to evaluate changes in cortico-muscular phase coherence to assess the effectiveness of rehabilitation strategies (i.e., passive vs. active, with or without exoskeleton or different level of engagement) and to serve as a biomarker for motor recovery in different pathologies [44,61,66,67,69,82]. In particular, the effect on CMC in post-stroke patients is mainly investigated [37,39,50,67,70,75,78]. However, the combination of EEG/EMG has demonstrated to efficiently evaluate the residual integrity of the neuromuscular system also in the spinal cord injury-affected subjects [38,63,65], in identifying low back pain-affected rehabilitation strategies [57] or to study the sensorimotor cortex in cerebral palsy-affected children [34]. As an example of stroke recovery evaluation, Chen et al. [81] demonstrated as a novel turning-based treadmill training was effective for enhancing brain functional reorganization underlying cortico-cortical and cortico-muscular mechanisms and thus might result in gait improvement in people with chronic stroke. Lai et al. [43] compared the outcome of functional electrical stimulation on 15 healthy subjects and 15 post-stroke patients and demonstrated that EEG-EMG coherence can detect electrical stimulation-induced changes in the neuromuscular system.

The literature describes other interesting applications based on other techniques of concurrent analysis. In Pierella et al. 2020 [33], the combined EEG/EMG analysis by PCA has shown the potential role to extract significant biomarkers for patient stratification as well as for the design of more effective rehabilitation protocols. Furthermore, the parameters extracted by the combined analysis of EEG and EMG signals can also be used to improve the classification of motor tasks in robotic rehabilitation if used to feed artificial intelligence approaches [68,87,88]. It is worth noticing that more advanced approaches, using Granger causality and PDC, were used to explore cortico-muscular connectivity and developed to detect complex functional coupling between cortical oscillations and muscle activities and provide a potential quantitative analysis measure for motion control and rehabilitation evaluation [40,42,45,46].

## 4. Discussion

In this systematic review, we analyzed papers in which EEG and EMG signals were simultaneously recorded and analyzed for evaluating or assessing motor performance in clinical rehabilitation scenarios.

From the distribution of selected papers over the years, the combined application of EEG and EMG signals to assess rehabilitation-related studies is in a growing trend. Indeed, most studies were published in the last decade with a remarkable increase in the last three years. This multi-domain approach was promoted by the improvement of technology and the availability of integrated low-cost commercial solutions aimed at combining EEG and EMG sensors. It arises from the literature that a multi-parametric analysis of EEG/EMG signals allows a more comprehensive investigation of complex neuromotor mechanisms with respect to exploiting a single technique. In fact, applying the two techniques independently cannot provide insight into mechanisms such as the functional connection between the central control system, the brain, and the actuators of movement, the muscles. Considering the EMG signal alone can provide information about muscles activation strategies, but no information can be inferred about the role of the cerebral control in it. Similarly, EEG signal alone during movement can provide information about the cerebral activation without control on the muscle-effective activation. These aspects can explain why the use of combined EEG/EMG analysis is rapidly becoming an emerging topic. All this finding supports the drafting of this review to summarize the results achieved so far.

In our sample of articles, we found that many studies were observational studies, mainly focused on the assessment of cortico-muscular coupling and the evaluation of the effects of treatment administration or rehabilitation methods, and pilot studies in which novel experimental setups or concept design were tested on a limited number of subjects. Only a few papers presented randomized clinical trials that evaluated the efficacy of rehabilitation paradigms by comparing the effects of rehabilitation interventions or treatments on a target group to a control one. The predominance of the pilot and observational studies indicated that the concomitant use of the techniques was found mainly in studies that aim at exploring novel research purposes rather than standard clinical practice. While this is perfectly understandable due to costs, invasive setups, time-consuming procedures, we conclude that, so far, the application and applicability of combined EEG-EMG setup is very limited in clinical practice.

It followed that these techniques were mostly employed in preliminary studies for evaluating the effects and efficacy of novel rehabilitation platforms and for proposing novel methodologies assessing motor performance. The exploratory design of the studies is also confirmed by the fact that in 45% of the analyzed papers, less than 10 subjects were enrolled. This shows their intrinsic nature as pilot studies. Future directions should foresee more structured studies; clinical trials could be developed starting from pilot studies already available so that more reliable conclusions on concomitant EEG-EMG applications can be drawn.

Moreover, EEG and EMG combined analysis was widely used for assessing functional connectivity and comparing cortico-muscular coherence in patients with respect to healthy subjects. Rarely was it used for evaluating clinical outcomes of motor rehabilitation, although it could provide a detailed assessment of patients’ status.

Indeed, many studies enrolled healthy subjects as a target for investigating physiological parameters concerning treatments or rehabilitation paradigms. The results obtained in healthy subjects can help to better understand the physiological mechanism underlying cortico-muscular activity and can be used as a benchmark for the pathological changes occurring in neuromotor disorders.

The pathologic subjects involved in the studies were mainly post-stroke patients, probably because stroke is one of the most diffuse cerebrovascular diseases affecting motor control. Other types of pathological conditions were investigated in a few or single articles. Future directions could be the extension of EEG-EMG combined analysis to neuromuscular pathologies that received little or less attention, such as neuromuscular diseases.

The integration of EEG and EMG signals can be useful for the evaluation of motor impairment and recovery, allowing the investigation of the motor system in its complexity. For example, the investigation of deficits of motor control exploits at best the potential of both EEG and EMG domains. Many studies exploit the cortico-muscular coherence, coupling the two domains in a single analysis [98]. Although this metric is the simplest approach to quantify the interactions between all motor system actors during movement, a more complex analysis could be of interest. There are very few studies that tend to exploit the full potential of each of the domains to unify data after assessment. In a recent paper [32], authors used domain-specific measurements (such as ERD-ERS for EEG and muscle synergies for EMG) and tried to interpret critically the detailed findings achieved in each domain. While some interpretations were still debated and not always fully agreed, authors could find a general agreement with other more easily interpreted data such as clinical scales and kinematics. A suggestion for future applications is thus to promote the use of advanced techniques for each of the domains under analysis. In fact, we also noticed that in many applications, EMG is seen as a supporting outcome measure for interpreting EEG data or even to simply allow signal synchronization through thresholding algorithms for detecting movement onset. Of course, while these approaches are perfectly scientifically sound, they might reduce the potential of advanced EMG analysis that allows refined measurements of motor control such as muscle synergies [90]. We also found that synthetic approaches suggested multimodal analysis as a tool for creating novel protocols and metrics based on the coupling of EEG and EMG (and other domains) [32,33]. The EEG/EMG bi-modal analysis, including also further domains, is an unexplored field that might help to shed light on the mechanisms of motor recovery.

For motor rehabilitation and assessment, a variety of experimental setups were explored in the analyzed papers. Many of them assessed motor function using only free movements coupled with sensorial feedbacks, motor imagery, or simple instruments. Robotics and exoskeletons are widely employed in motor rehabilitation to support and guide the movements of the patients preventing injuries and improving recovery using different loads and modes. Exoskeletons for assisting either upper limbs or lower limbs were quite diffused in literature, while robotic devices were found only for upper limb rehabilitation. Interestingly, while they have been used mainly for BCI setups [99,100], EEG and EMG may find wide application in the evaluation of human-robot interaction under a biomechanical perspective for assessment and evaluation.

Furthermore, particular attention was given to the rehabilitation of the hand because the upper limbs are the most affected in neuromotor disorders, and hand movements are involved in many daily life activities. Therefore, hand impairments limit heavily the ability to perform these activities [101,102], and motor recovery becomes essential for the patients’ quality of life. Different studies employed electrical stimulation, applying the stimulus either at the peripheral or at the cortical level. This application is useful for studying cortico-muscular connections in both healthy and pathological subjects. Moreover, functional electrical stimulation is demonstrated to be effective in improving muscle strength and motor coordination in patients [103,104].

Among all the papers included, different techniques were employed for the analysis of EEG and EMG signals, according to the aim of each study. EMG signal was mainly analyzed in the time domain, extracting indexes from muscle envelopes, while frequency analysis was used principally for evaluating muscle fatigue from the median frequency. Frequency and time-frequency analysis were predominant in EEG signal processing because the power spectrum can provide information about the mental and cognitive involvement of the subject [105]. Spatial distribution of the signals, as brain connectivity and muscle synergies, was assessed in only a few papers. However, studying the functional and effective connectivity of the brain can provide insight into the cortical reorganization and functional recovery of patients. Moreover, muscle synergies can be used to evaluate motor control and movement coordination that are affected in neuromuscular disorders. Therefore, including the analysis of the spatial distribution of the signals can provide further information about neuromotor impairment and motor improvement in rehabilitation. In this way, the potentiality of the instruments can be deeply exploited.

## 5. Conclusions

The evaluation of the complementary contribution of EEG and EMG signals to the assessment of cortico-muscular interactions in clinical rehabilitation of neuromotor diseases is a promising topic, and an increased number of applications and scenarios is foreseen in the next future. The combined analysis of EEG and EMG can be boosted by the development of consolidated pipelines, which warranties results in robustness and direct comparison among different studies, putting a special focus on the signal interactions in terms of functional and effective connectivity. Currently, the use of bi-modal EEG/EMG analysis helps to elucidate physiological and pathological mechanisms to assess the rehabilitation treatments and to evaluate their effectiveness. However, prospectively, multi-domain approaches should be developed to exploit the full potential of EEG and EMG, and more pathologies should be targeted with more structured clinical trials to improve the scientific evidence.

## Figures and Tables

**Table 1:** Type of study and aim.

Type of Study	Aim
Observational study	Study of the cortico-muscular coupling (1) during motor tasks [37,38,39,40,41] and (2) with electrical stimulation [42,43,44,45,46]
	Investigation of the effects of exoskeleton on functional connectivity [47,48,49]
	Investigation of the effects of visual feedback [50,51]
	Detection of movement intention [52,53] Study of the interhemispheric interaction with TMS [54]
	Study of a neurophysiological marker of stress [55]
	Study of slow cortical potentials in stroke [56]
	Study of correlation between lower back pain and altered postural stabilization [57]
Pilot study	Test new rehabilitation paradigm [34,58,59,60,61,62]
	Investigation of the efficacy of BMI [63] and EEG feedback [64]
	Study of movement classification combining EEG and EMG [65]
	Investigation of movement intention [66,67] and motor imagery detection [68]
	Study of the cortico-muscular coupling [69,70,71]
	Study of the neuroplasticity with electrical stimulation [72]
	Investigation of engagement in game rehabilitation [73]
	Study of the effects of the use of VR in facial rehabilitation [74]
Randomized controlled trial	Study the effects of transcranial [75,76] and peripheral electrical stimulation [77,78,79]
	Investigation of the efficacy of lower limb exoskeleton rehabilitation [80]
	Assessment of a novel gait training paradigm [81]
	Investigation of the efficacy of neurologic therapy based on music [82]
	Investigation of the effects of biofeedback [83]
Methodological study	Presentation of a multivariate approach for motor assessment [32,33] Presentation of a method for compressing EEG-EMG signal [84]
	Presentation of algorithms for motion detection [85,86] and motion classification [87,88]

**Table 2:** Subjects and anatomical targets.

Categories	Details and References	
Type of subjects	Healthy (Age range)	Target: 20–30 [61], n.a. [84], 24–36 [72], 60–62 [62], 18–35 [51], 57.8 ± 4.7 [66], 24 ± 2.32 [47,48], 23–27 [67], 26.86 ± 3.39 [52], 25.0 ± 1.7 [60], 22–28 [41], 22 [86], 22.8 ± 3.3 [87], 24.9 ± 5.4 [88], 21.2 ± 1.1 [44], 26.5 ± 6.5 [49], 23–27 [45,46]
		Control group: 20–39 [42], 53–62 [58], 27 ± 4 [38], 50.08 ± 15.8 [39], 35.4 ± 5.25 [57], 24 ± 1.5 [43], 27 ± 4 [65], 53 ± 14 [54], 58 ± 16 [33], 24–27 [40], 20 ≥ 60 [85], 33.5 ± 7.9 [53], 55.1 ± 2.1 [55], 35.9 ± 7.7 [68], n.a. [69,70], 42 ± 13 [71]
	Pathologic (Age range)	Chronic stroke: 53–72 [37], 37–72 [42], 35–63 [75], 61 ± 11 [32], 55–77 [80], 52–63 [58], 52.5 ± 9.7 [81], 56.5 ± 9.5 [39], 43–79 [43], 69.9 ± 10.5 [77], 46–81 [54], 56.5 ± 9.5 [78], 68 ± 18 [33], 52.7 ± 8.4 [74], 45–51 [40], 49.9 ± 10.9 [79], 56 [68], n.a. [70], 46–60 [71], 51.4 ± 11.1 [56]; Subacute stroke: 32–79 [50]; Subacute and chronic stroke: 52–64 [69], 42–92 [82]
		Parkinson disease: 40–80 [76]; Cerebral palsy: 5–19 [34]; Spinal cord injury: 32.5 ± 6.2 [38], 26–38 [63], 32 ± 6 [65], 43.5 ± 12.4 [53]; Writer’s cramp: 67 [64]; Low back pain: 39.2 ± 6.33 [57]; Mixed injuries and diseases: 33–54 [73], 60–80 [59]; Facial palsy: 23 [74]; MCI: n.a. [85]; Cardiovascular diseases: 56.3 ± 1.0 [55]
Nr of subjects	≤10	6 [37], 5 [32], 6 [34], 3 + 4 [58], 6 [61], 1 [84], 8 [63], 10 [72], 2 [62], 1 [64], 7 [66], 5 [47], 8 [67], 7 [52], 10 [33], 1 [74], 5 + 5 [40], 10 [41], 1 [86], 4 + 4 [53], 4 + 4 [71], 6 [49], 10 [45,46]
	10 < n ≤ 20	11 [75], 14 [50], 9 + 9 [81], 10 + 8 [38], 10 + 10 [57], 16 [51], 7 + 4 [69], 10 + 8 [65], 15 [77], 12 [78], 6 + 6 [33], 18 [87], 13 [44], 10 + 1 [68], 7 + 7 [70], 20 [56]
	>20	16 + 12 [42], 26 [76], 20 + 20 [80], 14 + 10 [39], 15 + 15 [43], 30 [47,48], 23 [59], 12 + 30 [82], 19 + 14 [54], 30 [83], 14 + 14 [79], 28 + 7 [85], 32 [88], 14 + 14 [55]
Rehabilitation target	Distal Upper Limb	Hand and wrist: [50,59,61,69,84]; Wrist: [37,40,44,75,82]; Hand: [39,43,45,46,51,54,56,62,64,67,73,76,78,83]
	Proximal Upper Limb	Shoulder and elbow: [32,33,58,60,70,71,82,84]; Elbow: [37,38,39,40,62,65,76,87,88]; Arm: [52,85,86]; Shoulder: [55]
	Distal Lower Limb	Ankle: [42,47,48,49,51,63,80]; Leg: [41,72,77,79,81]; Foot: [81]
	Proximal Lower Limb	Knee: [34,42,47,48,49,80]; Hip and knee: [63]
	Other	Torso: [57]; Face: [74]; Neck: [68]

**Table 3:** Setup for tests/rehabilitation.

Setup	Details and References
Miscellaneous techniques for free movements and rehabilitation	Mirror visual feedback [50]
	Wrist movements [69,84]; wrist movements + visual feedback [73]
	Elbow movements [38]; elbow movements + visual feedback [65]
	Arm movements [37,57,70,71,87,88]; arm movements + auditory feedback [82]; arm movements + balance handle [52]
	Hand movements [40,67,83,86]; hand movements + motor imagery + auditory/smelling stimulus [85]; hand movements + audiovisual feedback [56]; hand movements + biofeedback [64]
	Leg movements [53]
	Motor imagery swallow and tongue protrusion [68]
	Respiratory movements [55]
	Oculus rift [74]
Robotic assistance	Exoskeleton lower limb + virtual reality [34]; exoskeleton lower limb + virtual reality + BMI [63]; exoskeleton lower limb + walking [47,48,80]; exoskeleton lower limb + treadmill [49]
	Robotic end effector upper limb [32,33,58,59]
	Robotic mirror therapy upper limb [60]
	Hand mobilizer exoskeleton [39,61,62]; hand mobilizer exoskeleton + motor imagery [66]
	Ankle mobilizer [41]
Peripheral electrical stimulation	FES + hand movements [43,78]; FES + walking [79]; FES + hand movements + motor imagery [45,46]
	ePAS + ankle movements [77]
	NMES + wrist movements [44]
Transcranial electrical stimulation	HD-tDCs + wrist contractions [75]; anode tDCs + ankle dorsiflexion [72]
	rTMS [76]; TMS + wrist contractions [54]
Assisted rehabilitation	Pedaling system + NMES [42]
	Treadmill [81]; Treadmill + visual feedback [51]

**Table 4:** Setup for signal acquisition.

EEG ACQUISITION		
Setup	Details and References	
Number of electrodes	N > 100	163 [70,71]; 160 [58]; 128 [42,59];
	100 < N < 30	64 [32,33,34,38,44,50,65,66,75]; 62 [47,48]; 56 [37]; 40 [68,77]; 35 [67]; 32 [40,52,53,54,81,84];
	30 < N < 10	21 [39,80]; 20 [69,82]; 16 [43,49,56,63,78]; 15 [85]; 14 [62,73]; 10 [57,61,64];
	N < 10	8 [83]; 5 [72]; 3 [60,86,87,88]; 2 [45,46]; 1 [41,51,55,79];
Electrodes positioning	Motor area only [41,61,67]	
	Sensorimotor area [39,43,60,64,78]	
	Whole cortex [32,34,37,38,42,47,48,50,54,58,62,63,66,70,71,75,77]	
**EMG ACQUISITION**		
**Setup**	**Details and references**	
Number of electrodes	16 [53]; 15 [33,58]; 13 [71]; 10 [37,62]; 8 [32,56,70,73,76,80]; 6 [40,49]; 5 [57,63,84];	
	4 [38,39,42,47,48,51,59,65,68,69,75,81]; 3 [52,60,61,64,67];	
	2 [34,44,54,87,88]; 1 [41,43,55,66,72,77,78,79,82,83,85,86];	
Electrodes positioning	Single-joint	Wrist [43,44,50,54,59,61,62,64,66,67,73,75,78,81,83,85,86]
		Arm [38,55,60,65,82,87,88]
		Leg [34,72]
		Ankle [41,51,77,79]
		Face [74]
	Multi-joint	Lower limb [42,47,48,49,53,63,80]
		Upper limb [32,33,37,39,40,52,56,58,70,71,76,84]
		Trunk-upper limb [57]
	Multi-limb	Upper limb [37,70,71,76]

**Table 5:** Signal analysis techniques.

EEG Analysis Techniques	
Time domain analysis	ERP: [56,57,65,76,77]
	Cortical waves amplitude/slope: [53,74,87,88]
Frequency domain analysis	Average PSD: [34,41,42,47,49,52,59,65,72,75,79,83,86,87,88]
	Quantitative index calculation: [55,60,62,73]
Time-frequency analysis	ERD/ERS: [32,33,38,44,50,57,64,68,81]
	ERSP: [42,47,63]
Connectivity	Functional: [81,85]
	Effective: [48,54,80]
Sources reconstruction	ICA: [34,42,63]
	LORETA: [58,71,80]
**EMG Analysis Techniques**	
Time domain analysis	Amplitude/RMS: [34,55,58,59,60,63,73,74,77,79,80,83,86]
	Additional time features: [39,64,65,71,87,88]
Frequency domain analysis	Average PSD: [41,47,49]
	Median frequency: [44,60,79]
Time-frequency analysis	Channels coherence: [51]
	Time-frequency decomposition: [47]
Thresholding/landmark identification	EMG-EEG temporal synchronization: [54,57,62,72,81]
	Identification of events: [48,56,71,85,86]
Synergies	Non-negative matrix factorization algorithm: [32,33,53]
**EEG-EMG Combination**	
Cortico-muscular coherence	EEG-EMG coherence: [37,39,41,43,44,45,49,61,64,66,67,69,70,75,78,82,84]
	EEG-EMG PDC/gPDC: [42,46]
Time-frequency connectivity	Wavelet cross-spectrum: [38,51]
	Cross-mutual information: [66,81]
	Pearson’s correlation: [47]
	Copula Granger’s causality: [40]
Feature fusion for classification	Linear discriminant analysis: [65]
	Support vector machine: [87,88]

## Data Availability

Not applicable.
